# Prevalence and molecular characterization of drug-resistant *Mycobacterium tuberculosis* in Heyuan City in China

**DOI:** 10.3389/fcimb.2025.1586938

**Published:** 2025-06-12

**Authors:** Dan Qiu, JianHong Ma, ZhiFang Liu, XiangXing Zeng

**Affiliations:** ^1^ Medical Laboratory, Heyuan Key Laboratory of Molecular Diagnosis and Disease Prevention and Treatment, Doctors Station of Guangdong Province, Heyuan People’s Hospital, Heyuan, Guangdong, China; ^2^ Department of Traditional Chinese Medicine, Heyuan People’s Hospital, Heyuan, Guangdong, China; ^3^ Department of Clinical Laboratory, Heyuan People’s Hospital, Heyuan, Guangdong, China; ^4^ Department of Integrated Enforcement, Heyuan Health Supervision Institute, Heyuan, Guangdong, China

**Keywords:** drug resistance, gene mutation, MDR-TB, fluorescence melting curve analysis, Heyuan

## Abstract

**Purpose:**

Tuberculosis (TB) represents a significant global public health challenge, with China identified as a high-burden country. Data on the prevalence of drug resistance is crucial for informing the selection of appropriate pharmacological interventions for the treatment of drug-resistant tuberculosis (DR-TB).To evaluate the prevalence and drug resistance patterns among patients with DR-TB in Heyuan City, China.

**Methods:**

All 291 patients registered between April 2021 and March 2023 were tested for drug resistance, and information about their medical history and demographics was collected directly from the hospital’s computer database. Eight genes were analyzed for mutations associated with resistance to five antituberculosis drugs: the *katG*, *ahpC*, and *inhA* promoters for isoniazid (INH); *rpoB* for rifampicin (RIF); *embB* for ethambutol (EMB); *gyrA* for fluoroquinolones (FQs); and *rrs* and *rpsL* for streptomycin (STR). All strains were genotyped using fluorescence melting curve analysis.

**Results:**

In Heyuan, 24.4% (71/291) of patients with treatment-resistant TB were resistant to at least one drug. Following are the rates of general resistance to each drug: RIF (28/272, 10.29%), INH (38/274, 13.87%), FQs (10/259, 3.86%), EMB (20/248, 8.06%), and STR (15/150, 10.00%). Age or gender had no statistically significant impact on the likelihood of developing drug resistance. Nevertheless, a statistically significant difference was observed between the three strategies of drug resistance testing, AFB testing, and MTB antibody testing. There were 48 cases of single-drug resistance and 23 cases of multiple-drug resistance among the 71 drug-resistant patients. Eight genes had 127 altered nucleotide sequences, with KatG315 (20.47%) having the most significant incidence of mutations. The top three mutated genes were *rpoB* (32.28%), *katG* (23.62%), and *embB* (15.75%).

**Conclusion:**

These findings may be helpful in Heyuan City for the quick molecular identification of DR-TB isolates in clinical samples.

## 1 Introduction

Tuberculosis (TB), a contagious disease that significantly contributes to poor health and is one of the world’s leading causes of mortality, is caused by the transfer of *Mycobacterium tuberculosis* (MTB) through aerosols or droplets (Furin et al., 2019). In addition to causing TB in the host’s lungs, MTB produces secondary infections in the meninges, intestines, and peritoneum. The continuous evolution of MTB under the pressure of drug selection has facilitated the emergence of drug-resistant strains. Chromosomal single nucleotide polymorphisms (SNPs) or gene alterations mainly cause resistance to anti-TB medications (Liebenberg et al., 2022). It is now more likely that MTB will acquire resistance to the anti-TB drugs currently being used due to several variables, including the nature of the MTB cytoderm itself and the broad, continuous, and irregular use of anti-TB therapy (Heyckendorf et al., 2022; Liebenberg et al., 2022; Tiberi et al., 2022).

According to the World Health Organization’s (WHO) 2022 TB report (World Health Organization, 2022), there were 10.6 million new cases of TB in 2021, and 1.6 million TB-related fatalities were expected to be reported globally that same year, which is an increase of 4.5% and 100,000 deaths, respectively, compared to 2020. A total of 784,400 of those cases occurred in China (7.4%), one of the top three nations with a high incidence of TB, along with India (28%) and Indonesia (9.2%). The number of new patients with multidrug-resistant TB (MDR-TB) or Rifampicin-resistant TB (RR-TB) in 2021 was approximately 450,000 cases, while approximately 191,000 patients died from MDR-TB or RR-TB in the same year. The current global treatment success rate for DR-TB is 60% and remains low.

The situation regarding DR-TB prevention and control in China is similarly not optimistic. In 2021, China will have a population of 1.4 billion, with an estimated 784,000 new cases of TB (842,000 cases in 2020) and an estimated incidence rate of 55/10,000 cases of tuberculosis (59/10,000 cases in 2020). The number of patients with MDR/RR-TB is 33,000, with an incidence rate of 2.3/100,000, with 34% of new TB cases with MDR/RR-TB and 19% of previously treated TB cases with MDR/RR-TB. The traditional TB detection methods can no longer satisfy the country’s current TB prevention and control standards, particularly regarding the diagnosis and treatment of extensive and multidrug-resistant TB (MDR-TB). The WHO endorsed Xpert MTB/RIF technology in 2011 for speedy TB detection. However, it is expensive and only detects rifampicin mono-drug resistance (Shah et al., 2020; Wang et al., 2021). Fluorescence melting curve analysis (FMCA) is a real-time PCR qualitative diagnostic approach using a closed tube, two-color melting curve analysis, and dual-labeled self-quenching probes. It identifies the melting temperature (*T*_m_) at which MTB transforms from the wild type into the mutant. FMCA can simultaneously detect resistance to five drugs: rifampicin (RIF), isoniazid (INH), fluoroquinolones (FQs), ethambutol (EMB), and streptomycin (STR) (Hu et al., 2020).

Based on the WHO 2022 TB report, we can see that the geographical distribution of DR-TB evolves. DR-TB prevalence has not yet been identified in Heyuan City, China. Therefore, to enhance TB diagnosis and therapy, we analyzed the frequency and molecular characterization of drug resistance among DR-TB patients by using FMCA.

## 2 Materials and methods

### 2.1 Study population

Heyuan People’s Hospital serves as the sole municipal-designated medical institution for tuberculosis treatment in Heyuan City, admitting and treating nearly half of the city’s tuberculosis patients, and is mainly responsible for the management and treatment of tuberculosis patients in the region. This study conducts a retrospective analysis of 291 tuberculosis patients treated at Heyuan People’s Hospital from April 2021 to March 2023. Every registered case met the diagnostic requirements of the People’s Republic of China’s Diagnosis of TB in the Health Industry Standard for suspected cases, confirmed cases, and extrapulmonary tuberculosis. All participants or their families provided informed consent. The Medical Ethics Committee of the Heyuan People’s Hospital approved this study.

### 2.2 Testing for acid-fast bacilli

The anti-acid staining procedure was carried out in accordance with the kit manufacturer’s directions (Beso, Zhuhai, China), and a positive control was included. Oil microscopy revealed red rods and curved acid-resistant bacteria as signs of positive anti-acid staining.

### 2.3 MTB antibody testing

Blood samples from the selected patients were examined using the colloidal gold method to check for antibodies to MTB. Shanghai Oppo Biomedical Co. provided the equipment.

### 2.4 MTB DNA extraction

After a sputum sample had liquefied, 1 mL of the sample was centrifuged at 12,000 × g for 10 min, and the supernatant was discarded. Then, the sample was resuspended in 250 μL of TB DNA extraction solution by vortex shaking. The supernatant was transferred to a fully automated nucleic acid extraction tool (Zeesan, Xiamen, China) equipped with a DNA extraction kit, and the extraction liquid was used as a DNA template.

### 2.5 FMCA for the detection of drug resistance

FMCA was used to identify drug resistance for RIF, INH, EMB, STR, and FQs. All kits were from Xiamen ZhiShan Biotechnology Co. Detection of RIF resistance in the 81-bp RIF-resistance determining region of *rpoB* included codons 507–533. Mutations that conferred INH resistance were found in the promoter region of the *ahpC* loci (−44 to −30 nt and −15 to 3 nt), the codon of *inhA94*, the promoter region of the *inhA* loci (−17 to −8 nt), the *katG* gene deletion, and the *katG*315 codon. In codons 306, 406, and 497 of the *embB* gene, EMB resistance was discovered. Mutations in the *rpsL* gene at codons 43 and 88 and the *rrs* gene at loci 513–517 confer STR resistance. Codons 88–94 of the *gyrA* gene were used to identify FQ resistance. The PCR reactions were conducted in 25-μL systems. The PCR reactions and other experimental methods were carried out precisely as instructed, and the results were assessed in accordance with the guidelines included in the kit. Fluorescent signals for both FAM and TET channels were present in resistance mutation experiments for RIF, INH, EMB, and STR. In contrast, the FQ resistance mutation assay only excited one channel of the FAM fluorescence signal.

### 2.6 Data analyses

Data were processed using the statistical software SPSS 21.0. The statistical data were expressed as a rate (%), and the chi-squared (χ^2^) test was used. The Kappa consistency test was chosen to test different methods. *P*<0.05 indicates that the difference is statistically significant.

## 3 Results

### 3.1 Study population

**Figure 1:**
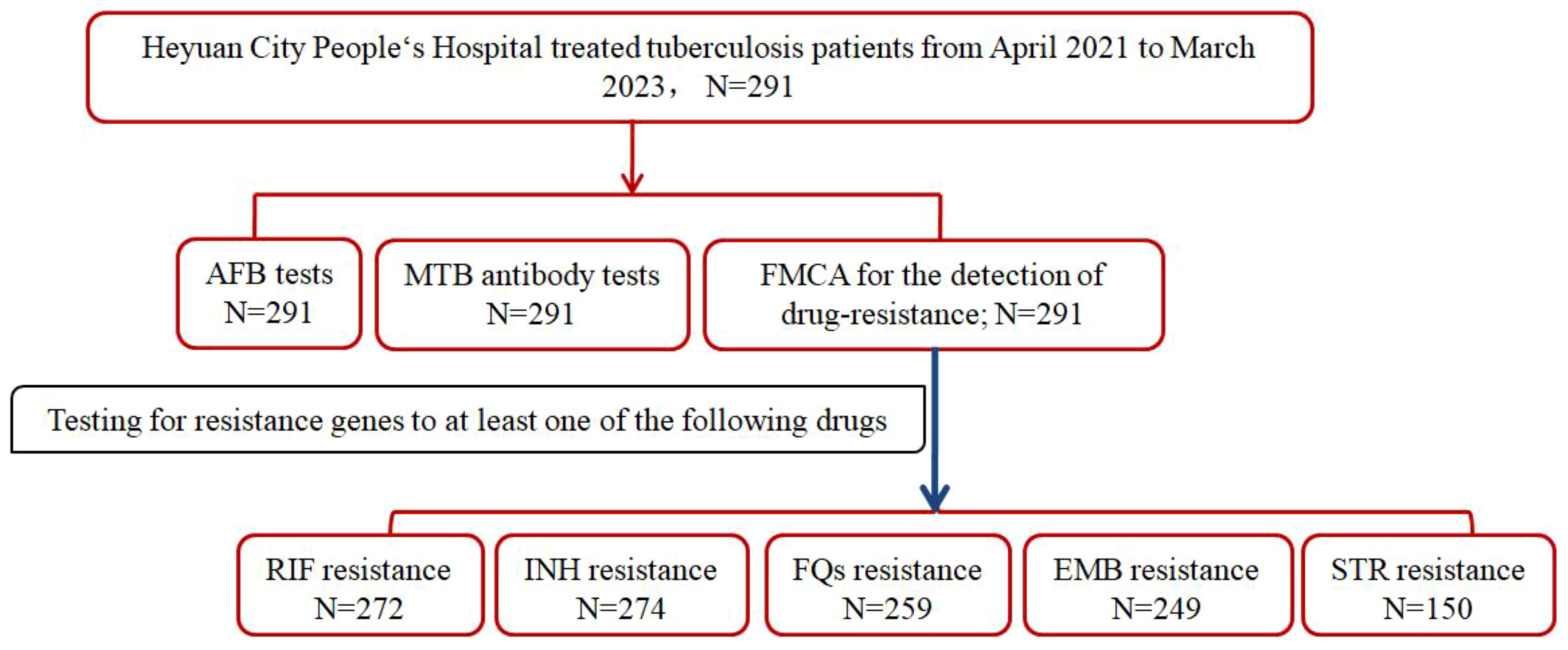
Study profile if DR-TB patients in Heyuan. MTBC, Mycobacterium tuberculosis complex; FMCA, Fluorescence Melting Curve Analysis; AFB, Acid-fast bacilli; DR-TB, drug-resistant tuberculosis.

In a cohort of 291 hospitalized patients, simultaneous assessments were conducted for acid-fast bacilli (AFB), Mycobacterium tuberculosis (MTB) antibodies, and resistance genes. Remarkably, resistance genes to at least one of the five drugs——RIF, INH, FQs, EMB, or STR—were detected in all patients. Specifically, resistance to RIF was evaluated in 272 patients, INH resistance in 274 patients, FQs resistance in 259 patients, EMB resistance in 249 patients, and STR resistance in 150 patients(**Figure 1**).

### 3.2 Comparison of different testing methods

The positive rates of the three different methods for the 291 specimens were as follows: 222 were positive and 69 were negative for the MTB antibody, or 76.3% (222/291); 153 were positive and 138 were negative for AFB, or 52.6% (153/291); and 71 were positive and 220 were negative for a drug-resistance mutation, or 24.4% (71/291). The three approaches had a positive detection rate for MTB, and the difference (*P*<0.001) was statistically significant. The kappa scores for the consistency analyses of all three of the methodologies were less than 0.1, indicating low consistency of the detection results.

### 3.3 Demographic characteristics

Of the 291 cases in the study, 228 (78.35%) were from male patients, and 63 (21.65%) were from female patients, with no statistically significant difference between the sexes. An age-wise analysis showed that patients with positive findings were aged 13–92 years, with a mean age of 52.7 years. Further statistical analysis based on age showed no statistically significant difference between the age groups. There were 243 newly diagnosed cases (243/291, 83.51%), which was a large proportion. In addition, there were 33 (11.34%) repeat treatment cases and 15 (5.15%) cases in which previous treatment had failed. Repeat treatment cases had a higher probability of drug resistance than did newly diagnosed cases or cases of forward treatment failure (*P*<0.05). The detailed demographic characteristics are shown in **Table 1**.

**Table 1:** Demographic characteristics of 291 cases of tuberculosis in Heyuan.

Characteristics	Number of isolates (n=291)	Number (%) of isolates			*P*-value
		Resistant ^a^	Susceptible ^b^	χ2	
Sex					
Male	228 (78.35%)	59 (83.10%)	169 (76.82%)	1.248	0.264
Female	63 (21.65%)	12 (16.90%)	51 (23.18%)		
Age					
≤29	43 (14.78%)	9 (12.68%)	34 (15.45%)	8.856	0.115
30~39	31 (10.65%)	4 (5.63%)	27 (12.27%)		
40~49	47 (16.15%)	9 (12.68%)	38 (17.27%)		
50~59	63 (21.65%)	18 (25.35%)	45 (20.45%)		
60~69	53 (18.21%)	11 (15.49%)	42 (19.09%)		
≥70	54 (18.56%)	20 (28.17%)	34 (15.45%)		
Treatment history					
Newly diagnosed case ^c^	243 (83.51%)	57 (80.28%)	186 (84.55%)	17.342	0.000
Retreatment cases ^d^	33 (11.34%)	4 (5.63%)	29 (13.18%)		
Previously treated failure cases ^e^	15 (5.15%)	10 (14.08%)	5 (2.27%)		

a The isolated strain is resistant to one of the drugs: RIF, INH, FQs, EMB, and STR.

b The isolated strain is susceptible to all drugs: RIF, INH, FQs, EMB, and STR.

c Newly diagnosed case: test cases for drug resistance for the first time.

d Retreatment cases: who was once cured but relapsed or were treated more than one month but treatment interruption was longer than two months; now accept treatment again.

e Previously treated failure cases: still sputum smear-positive after the whole treatment (6 months) or treatment for five months.

### 3.4 Molecular characterization

**Figure 2:**
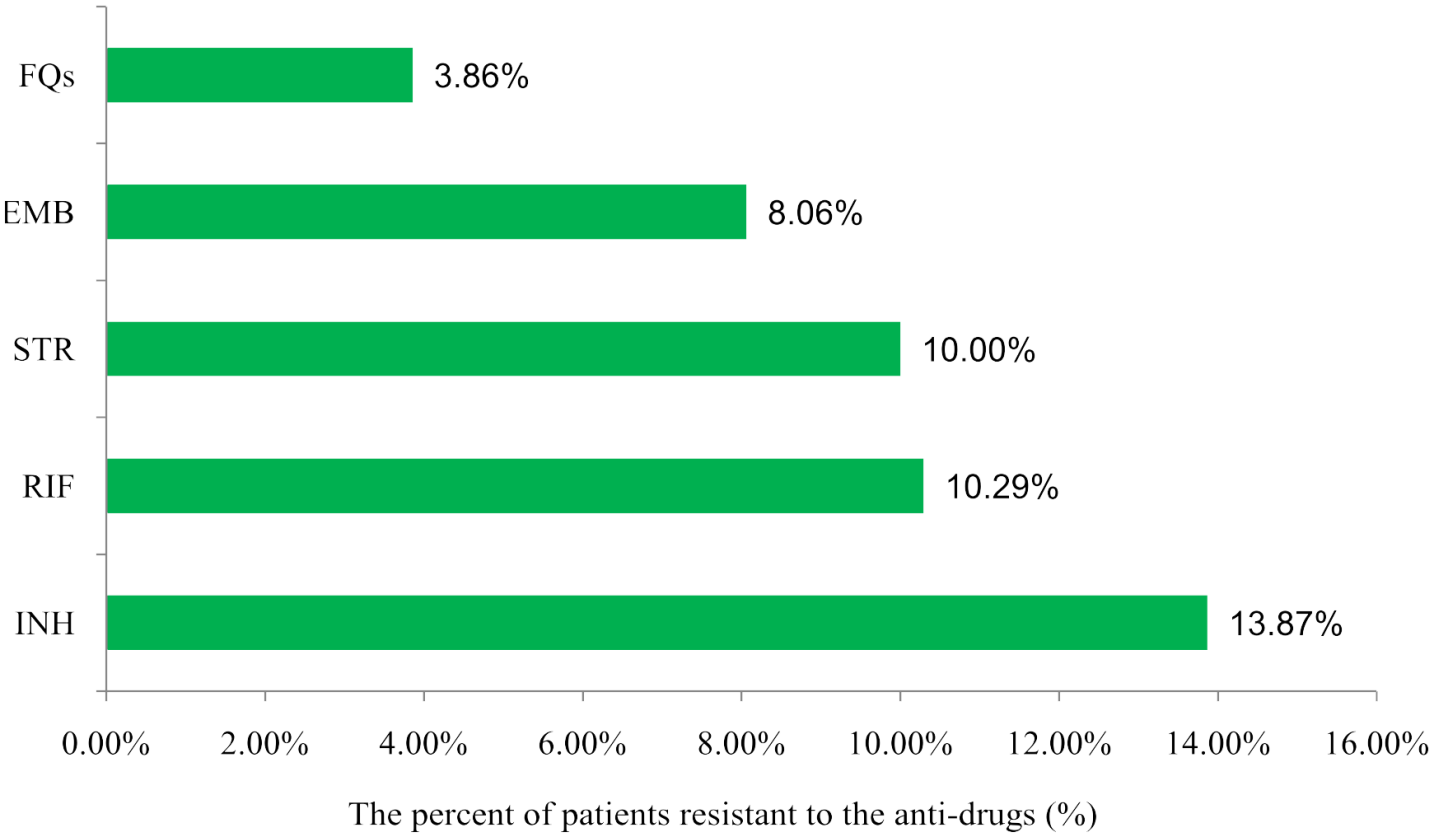
Distribution of drug resistance among TB patients to different anti-drugss in Heyuan. RIF, rifampicin; INH, isoniazid; FQs, flouroquinolones; EMB, ethambutol; STR, streptomycin.

In the 291 positive samples, 71 isolates(24.4%, 71/291)were resistant to at least one of the five anti-TB drugs, including 48 isolates that were one drug-resistant, 12 isolates that were two drug-resistant, eight isolates that were three drug-resistant, and three isolates that were five drug-resistant. The results are shown in **Table 2**. In order to determine this, we added the number of monoresistant and multiresistant isolates and calculated how many cases were resistant in relation to the 291 positive cases. The overall rate of resistance to each drug was as follows: RIF (28/272, 10.29%), INH (38/274, 13.87%), FQs (10/259, 3.86%), EMB (20/248, 8.06%), and STR (15/150, 10.00%) (**Figure 2**). Variations in the resistance rates to the five antimicrobial medicines were statistically significant (χ^2^) = 46.777, *P*<0.001).

**Table 2:** Patterns of drug resistance in 71 cases of TB patients in Heyuan.

Drug	Number (n=71)	Proportion (%)
Resistant single drug	48	67.61%
INH	18	25.35%
RIF	13	18.31%
STR	7	9.86%
EMB	6	8.45%
FQs	4	5.63%
Resistant two drugs	12	16.90%
INH+EMB	4	5.63%
INH+STR	2	2.82%
RIF+INH	2	2.82%
RIF+EMB	2	2.82%
RIF+STR	1	1.41%
INH+FQs	1	1.41%
Resistant three drugs	8	11.27%
RIF+INH+EMB	5	7.04%
RIF+INH+FQs	1	1.41%
RIF+INH+STR	1	1.41%
INH+FQs+STR	1	1.41%
Resistant five drugs	3	4.23%
RIF+INH+FQs+EMB+STR	3	4.23%

RIF, rifampicin; INH, isoniazid; FQs, fluoroquinolones; EMB, ethambutol; STR, streptomycin.

### 3.5 Genotyping of DR-TB strains

The 71 drug-resistant strains included 127 mutated loci or gene regions in eight genome regions linked to drug resistance. The *rpoB* gene had the highest percentage of mutations (32.28%), followed by the *katG* (23.62%), *embB* (15.75%), *gyrA* (7.87%), *rpsL* (7.08%), *inhA* (5.51%), *rrs* (4.72%), and *ahpC* (3.14%) genes. The top five mutated nucleotide sequences, accounting for 20.47%, 9.45%, 8.66%, 7.87%, and 7.87% of the eight genes detected, were *katG* 315, codons at 529–533 of the *rpoB* gene, *rpoB* codons at loci 521–528, codons at 507–512 of the *rpoB* gene, and codons at 88–94 of the *gyrA* gene, respectively (**Table 3**).

**Table 3:** Analysis of drug resistance and genotype in 71 patients in Heyuan.

Type of drug resistance	Gene	Locus of mutation	Number(%)	The proportion of each gene mutation	Total
RIF	*rpoB*	Codons at loci 529 to 533	12(9.45%)	32.28%	32.28%
		Codons at loci 521 to 528	11(8.66%)		
		Codons at loci 507 to 512	10(7.87%)		
		Codons at loci 513 to 520	8(6.3%)		
INH	*katG*	Codon at 315	26(20.47%)	23.62%	32.28%
		katG gene deletion	4(3.15%)		
	*inhA*	Promoter region -17 to -8 loci	6(4.72%)	5.51%	
		Codon at 94	1(0.79%)		
	*ahpC*	Promoter region -44 to -30 loci	2(1.57%)	3.14%	
		Promoter region -15 to 3 loci	2(1.57%)		
EMB	*embB*	Codon at 306	8(6.30%)	15.75%	15.75%
		Codon at 368/378/380	6(4.72%)		
		Codon at 406	4(3.15%)		
		Codon at 497	2(1.57%)		
FQs	*gyrA*	Codons at loci 88 to 94	10(7.87%)	7.87%	7.87%
STR	*rpsL*	Codon at 43	6(4.72%)	7.08%	11.81%
		Codon at 88	3(2.36%)		
	*rrs*	Mutations at loci 905 to 908	4(3.15%)	4.72%	
		Mutations at loci 513 to 517	2(1.57%)		
		Total	127(100%)	100.00%	

RIF, rifampicin; INH, isoniazid; FQs, fluoroquinolones; EMB, ethambutol; STR, streptomycin.

## 4 Discussion

Although several studies have examined the prevalence of MTB mutations causing drug resistance, this is the first study to describe changes in eight genes associated with drug resistance in Heyuan City, Guangdong, China. Due to factors including the variety of the TB pandemic and the actual usage of antimicrobial drugs, the incidence of MDR-TB might differ from one location to another (Dean et al., 2022). In this investigation, 24.4% of patients with drug-resistant TB in Heyuan were resistant to at least one drug, slightly higher than in Jiangxi (18.08%) (Luo et al., 2019) and lower than in Beijing (60.58%) (Yin et al., 2022) and Hunan (40.9%) (Zhao et al., 2014), and closer to Hainan (24.9%) (Liu et al., 2021). In this study, 67.61% (48/71) of patients with drug resistance were monoresistant, indicating that they were only one step away from developing multidrug-resistant TB. It is, therefore, imperative that appropriate treatment regimens are advocated in order to prevent the spread of MDR-TB.

Research has demonstrated that base mutations in some regions of genes encoding drug targets or enzymes linked to drug action on chromosomes are the primary cause of the development of drug resistance in MTB. The diagnosis of conventional tuberculosis relies fundamentally on the presence of AFB cultures and phenotypic drug susceptibility tests (Liebenberg et al., 2022). However, as MTB cultures take a long time to produce results, clinicians do not have results to use when they begin empirical treatment. FMCA is one of the most convenient and reliable methods for analyzing genotypes (Dong et al., 2022). The three tests in this study’s comparative analysis revealed poor consistency. However, FMCA still deserves consideration and use due to its quick detection time and high specificity (Hu et al., 2020).

The emergence of treatment resistance in TB is typically correlated with demographic traits, including medical problems and socioeconomic variables (Singh and Chibale, 2021). Heyuan is a city in northeastern Guangdong Province. The results of this study showed that neither gender nor age was statistically significant in terms of the risk of drug resistance, contradicting findings from both a Chinese report (Luo et al., 2019; Yin et al., 2022) and a European study (Faustini et al., 2006). The exact cause of this discrepancy is unknown, but it may be related to the small sample size of this study and the locals’ way of life. Retreatment cases were found to have a higher risk of drug resistance than cases that had failed treatment before (*P*<0.01). Patients with more prolonged treatment histories were more likely to have isolates resistant to medication in the retreatment case group. This relationship may, in part, be a result of ineffective medication management in healthcare facilities.

Among the 243 newly confirmed cases of infection, 57 drug-resistant MTB strains were discovered, showing a significant prevalence of drug-resistant strains in Heyuan, which increases the complexity of treating TB. However, the specific details of its prevalence still need to be clarified through further research.

Numerous studies have demonstrated that mutations in the 81-bp core region of the *rpoB* gene are crucial for predicting phenotypic RIF resistance and account for more than 95% of RIF resistance (Mathiasen et al., 2019). In this study, 28 out of the 71 resistant strains exhibited mutations in the *rpoB* gene core region, as detailed in **Table 2**, which includes 13 mono- and 15 multi-resistant strains. The *rpoB* gene core region mutations accounted for 32.28% of the total mutation loci or gene region, much lower than the percentage of *rpoB* gene mutations found in Jiangxi (Luo et al., 2019). Studies have shown that INH resistance occurs in conjunction with RIF resistance. Therefore, RIF resistance is sometimes a more accurate indicator of MDR-TB (Mathiasen et al., 2019). In this study, a total of 38 patients with INH resistance were identified, including 18 cases of monoresistance and 20 cases of multi-drug resistance. The central locus of INH resistance in Heyuan was *KatG315* (20.47%). The proportion of mutations at the *KatG315* locus ranged from 55.5% in the Western Pacific region to 78.4% in Southeast Asia (Narmandakh et al., 2020). The *inh*A promoter region was the second most commonly mutated region, identified in 4.72% of INH phenotypic resistance cases.

Our investigation discovered 20 and 15 instances of drug resistance observed in EMB and STR, respectively, with six and seven cases of single resistance and 14 and eight samples of multiple resistance. In the *embB* gene, codon 306 mutations were the most frequent mutation conferring EMB resistance in our study, similar to that of Ningbo (Che et al., 2022). Resistance genes *rrs* and *rpsL* have been found to be linked to STR resistance, and encode 16srRNA and ribosomal protein S12, respectively. In our study, mutations in th*e rpsL ge*ne were predominant among STR-resistant isolates. FQs primarily affect DNA gyrase to prevent DNA replication, which kills bacteria. The *gyrA* and *gyrB* genes encode two A and two B subunits of the DNA gyrase to form a tetramer. According to reports, *gyrA* gene mutations in MTB are strongly associated with FQ resistance. *gyrB* gene changes are infrequently associated with treatment resistance (Sun et al., 2008; Zhang et al., 2014). Therefore, in this investigation, the *gyrA* gene was the only subunit analyzed.

Although we achieved several meaningful findings in the present study, there are items that still need attention. Even though the current study yielded several essential conclusions, specific issues still require addressing. First, we chose the FMCA method for detecting drug resistance genes. This method enables rapid identification of both MTB and non-MTB. It is less affected by the quantity and purity of the bacteria in the specimen. However, because the known resistance genes are not sufficiently comprehensive, this method may under-detect resistance genes by only detecting known resistance loci or mutated fragments. This technique also does not pinpoint the precise SNPs that cause resistance. Second, our ability to detect drug resistance gene alterations and TB drug resistance may have been hampered by the fact that we only collected patient sputum for examination, disregarding such other specimens as alveolar lavage fluid. Finally, there may be bias in our analysis of TB resistance and multi-drug resistance because we primarily screened for first-line antimicrobials and did not perform drug sensitivity testing for second-line anti-TB medications in any of the multidrug-resistant patients.

## 5 Conclusion

This research has shown that 24.4% of DR-TB patients in Heyuan were resistant to at least one TB drug. The *katG315* mutation was the most frequent mutation in Heyuan, consistent with results from other regions worldwide. Furthermore, neither age nor gender had a statistically significant impact on the likelihood of drug resistance. This is inconsistent with the results in some areas of China and needs further exploration. Meanwhile, drug resistance testing of anti-tuberculosis drugs should be further expanded in the follow-up work, so as to provide a more comprehensive data reference for tuberculosis prevention and treatment.

## Data Availability

The original contributions presented in the study are included in the article/supplementary material. Further inquiries can be directed to the corresponding author.
